# Cavernous Transformation of the Portal Vein in a 26-Month Old Boy Treated by Transjugular Intrahepatic Portosystemic Shunt: A Case Report

**DOI:** 10.3389/fped.2019.00379

**Published:** 2019-09-18

**Authors:** Bo Wei, Linhao Zhang, Huan Tong, Zhidong Wang, Hao Wu

**Affiliations:** ^1^Department of Gastroenterology, West China Hospital, Sichuan University, Chengdu, China; ^2^Laboratory of Gastroenterology and Hepatology, West China Hospital, Sichuan University, Chengdu, China

**Keywords:** cavernous transformation of portal vein, transjugular intrahepatic portosystemic shunt, variceal bleeding, young children, case report

## Abstract

Cavernous transformation of portal vein (CTPV) is the main cause of portal hypertension and its related variceal bleeding in children. Transjugular intrahepatic portosystemic shunt (TIPS) was not reported to treat CTPV for children younger than 5 years old. In this case report, the patient was a 26-month-old boy who presented with hematemesis and melena due to CTPV. Even after azygoportal disconnection, repeated hematemesis as well-melena still occurred. After careful evaluation, we performed TIPS under general anesthesia for him. The procedure was uneventful, and 6-mm stents were inserted. Six months after TIPS, there was no recurrence of bleeding, and no procedure-related event happened. The follow-up esophagogastroduodenoscopy proved dramatic remission of varices, indicating a successful outcome. We believe TIPS could be safely placed for young children to manage variceal bleeding due to CTPV.

## Introduction

Cavernous transformation of the portal vein (CTPV) is commonly known as the replacement of the obstructed portal vein by numerous tortuous venous channels. In children, CTPV is the main cause of portal hypertension (1, 2). Despite its rarity (prevalence <5/10,000), the resultant portal hypertension could cause life-threatening variceal bleeding, which usually initiates before 10 years of age (3). There are many treatment strategies, but transjugular intrahepatic portosystemic shunt (TIPS) is minimally invasive and, most importantly, could alleviate portal hypertension directly. Though TIPS placement has been described as being able to treat portal hypertension in children (4), due to its technical difficulty and the small size of young children, to date, no report has been published delineating TIPS as the treatment for pediatric CTPV under the age of 5 years of age. Herein, we describe the first case of CTPV in a 26-month old boy treated by TIPS.

## Case Report

A 26-month-old boy was admitted to our hospital due to repeated melena and hematemesis. Eight months prior, intermittent hematemesis as well as melena initiated due to CTPV, diagnosed in another hospital. Six months prior, azygoportal disconnection without splenectomy was performed to control the symptoms. However, 5 months prior, melena recurred and haematemesis happened 3 times afterwards till readmission. His previous medical history included esophageal atresia (cured previously in another hospital by surgery) and atrial septal defect (untreated due to good cardiac function). Umbilical cord infection was ruled out.

**Figure 1:**
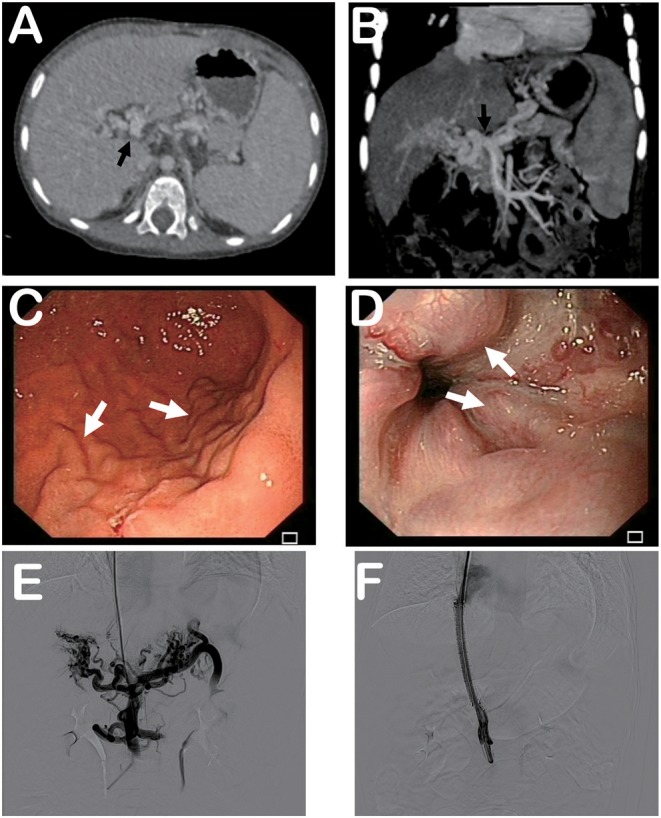
Computed tomography, esophagogastroduodenoscopy, and portography. **(A)** Computed tomography showing splenomegaly and signs of cavernous transformation of portal vein. **(B)** Tomographic reconstruction showing cavernous transformation of portal vein. **(C)** Esophagogastroduodenoscopy demonstrating gastric varices. **(D)** Esophagogastroduodenoscopy revealing esophageal varices. **(E)** Portography before stent placement indicating formation of multiple convolute collateral vessels at hepatic hilus. **(F)** Patency of portal vein and stent was confirmed by portography after stent placement. Black arrows indicate portal vein. White arrows indicate varices.

On admission, the patient was stable. His heart rate was 95 bpm and body temperature was 36.5°C. His height was 90 cm (*z*-score 0.645) and body weight was 12.4 kg (*z*-score 0.000). Relevant laboratory results are listed in Table 1. Computed tomography (CT) found esophageal varices and splenomegaly without ascites (Figure 1A). Branches of portal vein were not distinguishable and replaced by multiple convolute collateral vessels, which was consistent with features of CTPV (Figures 1A,B). Esophagogastroduodenoscopy demonstrated grade 3 gastroesophageal varices (Figures 1C,D).

**Table 1:** Laboratory results on admission.

**Variables**	**On admission**	**6 months post-TIPS**	**Reference ranges**
Red blood cell (×10^12^/L)	3.18	4.36	3.9–5.4
Hemoglobin (g/L)	84	119	110–145
Platelet count (×10^9^/L)	54	159	100–300
Total bilirubin (μmol/L)	7.7	10.2	5.0–25.0
Direct bilirubin (μmol/L)	1.8	3.0	<6.8
ALT (U/L)	31	35	<45
AST (U/L)	43	51	<75
Albumin (g/L)	33.2	40.8	35.0–55.0
APTT (s)	34.7	41.7	28.0–42.0
PT (s)	15.4	21.8	11.5–15.0
INR	1.18	1.94^†^	0.80–1.50

† *The increased INR is due to warfarin intake. We prescribed warfarin for this patient after shunt creation because he had 4G/5G plasminogen activator inhibitor-1 gene polymorphism, which indicated that he was under high risk for thrombosis*. *ALT, alanine aminotransferase; AST, aspartate aminotransferase; APTT, activated partial thromboplastin time; INR, international normalized ratio; PT, prothrombin time*.

In view of poor response of surgery and after careful consideration, TIPS under general anesthesia was thought to be the safest option and could relieve portal hypertension.

Before TIPS, his CT scan was evaluated to make sure that Rupus-100 (Cook Incorporated, Bloomington, IN, USA), the TIPS kit for adults, was accessible to his vasculature since pediatric TIPS sets are lacking. After general anesthesia, his right internal jugular vein was punctured. Access to the portal vein from the right hepatic vein was guided by X-ray. Portography revealed that the main portal vein at the hepatic hilus was occluded entirely with formation of multiple convolute collateral vessels. Also, splenic vein flowed hepatofugally, and esophageal varices were evidenced (Figure 1E). The portosystemic pressure gradient was 22 mmHg. Because the portal vein diameter was measured to be 9 mm, a 6 ×60 mm covered Fluency stent (Bard Incorporated, Karlsruhe, Germany) and a 6 ×60 mm bare SMART stent (Cordis, FL, USA) were inserted between the hepatic vein and portal vein after dilatation of the intrahepatic tract. Patency of portal vein and stent was confirmed by portography afterwards (Figure 1F), and the portosystemic pressure gradient was reduced to 6 mmHg. Recovery from anesthesia was uneventful. His condition was stable and he was discharged 3 days after TIPS placement.

**Figure 2:**
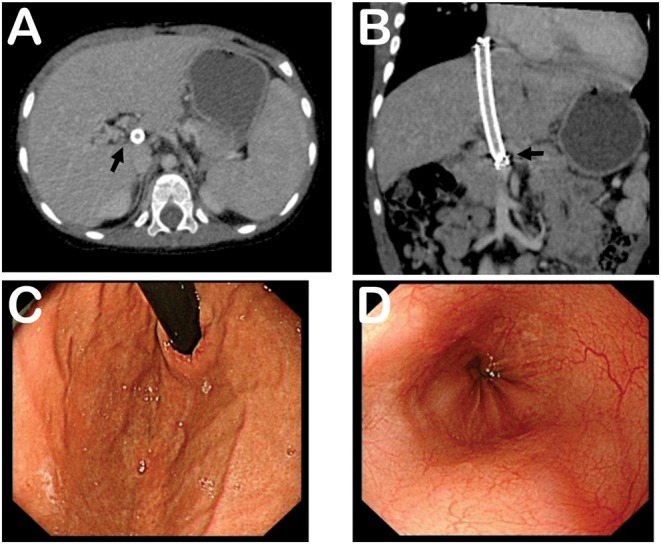
Follow-up computed tomography and esophagogastroduodenoscopy at 6 months post-TIPS. **(A)** Computed tomography showing shunt and smaller size of spleen. **(B)** Tomographic reconstruction showing stent and portal vein. **(C)** Esophagogastroduodenoscopy demonstrating relieved gastric varices. **(D)** Esophagogastroduodenoscopy revealing relieved esophageal varices. Black arrows indicate stent and portal vein.

Six months post-TIPS, relapse of melena and hematemesis, and occurrence of hepatic encephalopathy were not noticed. Relevant follow-up laboratory results are listed in Table 1. The shunt functioned well as determined by ultrasound and CT. The size of the spleen was smaller and the varices were relieved dramatically, determined by CT and the esophagogastroduodenoscopy (Figure 2). Furthermore, his development was not impaired. His height increased to 95 cm (*z*-score 0.912), which was 5 cm taller than 6 months before. Body weight increased to 14.5 kg (*z*-score 0.750), which was 3 kg heavier than 6 months before. The growth level and velocity were both within normal range. Language development was also normal.

## Discussion

In 1997, TIPS was used for the first time to control variceal bleeding in 10-month-old infant with biliary atresia (5). Since then, several reports have been published, and TIPS was used to deal with biliary atresia, cystic fibrosis, autosomal recessive polycystic kidney disease, Budd-Chiari Syndrome, and extrahepatic portal venous obstruction (including CTPV) in children, mainly indicated for variceal bleeding due to portal hypertension (2, 6–9). However, to our knowledge, TIPS was not reported to treat CTPV in children under the age of 5 years old. To treat CTPV with TIPS is technically difficult, and the success of our case suggested that even for a boy as young as 26 months old, its effect could be promising.

The treatment strategies to CTPV include medication, endoscopic management (sclerotherapy and band ligation), surgery (splenectomy, surgical shunt, and liver transplantation), and TIPS (1, 6). Though TIPS is considered challenging in the presence of CTPV, we deemed TIPS optimal compared with other strategies for several reasons, as listed in Table 2. In brief, for this particular patient, his portal hypertension should be managed directly to prevent further bleeding events. In this way, the Meso-Rex-Shunt seemed to be a good choice but repeated surgery should be avoided as he received operations before, otherwise abdominal adhesion (adhesive fibrosis) and other complications might occur (10). Most importantly, the boy's parents denied surgery and preferred minimally invasive methods to deal with the issue. Thus, after careful evaluation, TIPS was finally chosen as the therapy. Its minimal invasiveness and the direct effect on reducing portal hypertension were both favored for our patient.

**Table 2:** Pros and cons of different therapeutic regimens.

**Therapeutic regimens**	**Pros**	**Cons**
Endoscopic management (sclerotherapy & band ligation)	1. Minimally invasive. 2. Relieve varices.	1. Portal hypertension could not be resolved. 2. Bleeding might recur.
Splenectomy	1. Relieve varices. 2. Might relieve portal hypertension partially.	1. Repeated surgery. 2. Might induce portal vein thrombosis. 3. Might lead to adverse immune function and cause infection afterwards.
Liver transplantation	Relieve portal hypertension.	1. Not cost-effective due to good liver function. 2. Waiting time too long. 3. Repeated surgery. 4. Technically difficult considering portal cavernoma.
Surgical shunt	Relieve portal hypertension.	1. Repeated surgery. 2. Might lead to abdominal adhesion and other complications.
TIPS	1. Minimally invasive. 2. Relieve portal hypertension.	1. Technically difficult. 2. Shunt might occlude during follow-up period.

*TIPS, transjugular intrahepatic portosystemic shunt*.

However, to perform TIPS for this boy was difficult. First, the small size of the boy was challenging. No pediatric TIPS set is available, so we repeatedly confirmed that Rupus-100 was accessible to his internal jugular vein and hepatic vein. Due to the small size of his liver, caution was also exerted to prevent puncture into the abdominal cavity. Second, due to the cavernous transformation, identifying the portal vein was difficult. Thus, indirect portography was performed by injection of contrast medium to superior mesenteric artery. In this way, the upper part of the residual portal trunk, which was adjacent to liver parenchyma, was selected to be accessed. This was because the diameter of the portal trunk was large enough and access to other branches of extrahepatic portal vein might lead to intraabdominal hemorrhage. Third, the selection of the stent should be cautious for small children. We measured that his portal vein was 9 mm in diameter, and thus an 8 mm stent could be too large. Thus, 6 mm stents were chosen, which should be regarded as appropriate because the boy did not suffer from hepatic encephalopathy till now.

The outcome was successful. Six months after shunt creation, he did not suffer from relapse of melena and hematemesis or complications due to TIPS procedure. Esophagogastroduodenoscopy also demonstrated that varices were relieved. Moreover, his growth and development were also unimpaired. In particular, hepatic encephalopathy was not observed during follow-up. According to several studies, incidence of hepatic encephalopathy after TIPS for pediatric patients seemed to be lower than adults, ranging from 0 to 15% during the follow-up period up to 104 months (2, 11–13). Lower rate of hepatic encephalopathy might be attributed to prehepatic portal hypertensive etiologies without liver cirrhosis (as in this case) in pediatric patients (2, 11). However, another study suggested that nearly half of their pediatric patients undergoing TIPS would develop hepatic encephalopathy (14). Intriguingly, the authors in this study suggested that this high rate of hepatic encephalopathy might be related with medical comorbidities and over-dilation of shunts in their cohort (14). Nevertheless, all these reported hepatic encephalopathy had been successfully managed with medication (2, 11–14). It seemed that hepatic encephalopathy might be a minor problem after TIPS in pediatric patients compared with that in adults.

In conclusion, we reported the first case of CTPV under the age of 5 successfully treated by TIPS. We believe TIPS may represent an effective alternative in reducing the sequelae of portal hypertension in order to prevent variceal bleeding in individual cases of young children.

## Data Availability Statement

The datasets generated for this study are available on request to the corresponding author.

## Ethics Statement

Ethical review and approval was not required for the study on human participants in accordance with the local legislation and institutional requirements. Written informed consent to participate in this study was provided by the participants' legal guardian/next of kin.

## Author Contributions

BW: performed TIPS, revised the manuscript, and approved the final manuscript. LZ: participated in clinical assistance of the patient, collected and interpreted the clinical data, reviewed the literature, drafted the manuscript, and approved the final manuscript. HT: collected and interpreted the clinical data, helped draft the manuscript, and approved the final manuscript. ZW: participated in clinical assistance of the patient, searched the literature, and approved the final manuscript. HW: conceived this study, confirmed the disease, performed TIPS, revised and reviewed the manuscript, and approved the final manuscript.

### Conflict of Interest

The authors declare that the research was conducted in the absence of any commercial or financial relationships that could be construed as a potential conflict of interest.

## References

[1] Young V Rajeswaran S. Management of portal hypertension in the pediatric population: a primer for the interventional radiologist. Semin Intervent Radiol. (2018) 35:160–4. 10.1055/s-0038-1660794 30087518 PMC6078695

[2] Lv Y He C Guo W Yin Z Wang J Zhang B. Transjugular intrahepatic portosystemic shunt for extrahepatic portal venous obstruction in children. J Pediatr Gastroenterol Nutr. (2016) 62:233–41. 10.1097/MPG.0000000000000982 26381818

[3] Shneider BL de Ville de Goyet J Leung DH Srivastava A Ling SC Duche M. Primary prophylaxis of variceal bleeding in children and the role of MesoRex Bypass: summary of the Baveno VI Pediatric Satellite Symposium. Hepatology. (2016) 63:1368–80. 10.1002/hep.28153 26358549

[4] Tabriz DM Lazarowicz MP Beasley GL Toskich BB. Transjugular intrahepatic portosystemic shunt creation in a 5.5-kg infant with refractory variceal hemorrhage: case report and review of the literature. J Vasc Interv Radiol. (2016) 27:145–8. 10.1016/j.jvir.2015.09.020 26723926

[5] Cao S Monge H Semba C Cox KL Berquist W Concepcion W. Emergency transjugular intrahepatic portosystemic shunt (TIPS) in an infant: a case report. J Pediatr Surg. (1997) 32:125–7. 10.1016/S0022-3468(97)90117-2 9021592

[6] Sorrentino D Labombarda A Debiase F Trevisi A Giagu P. Cavernous transformation of the portal vein associated to multiorgan developmental abnormalities. Liver Int. (2004) 24:80–3. 10.1111/j.1478-3231.2004.00890.x 15102004

[7] Verbeeck S Mekhali D Cassiman D Maleux G Witters P. Long-term outcome of transjugular intrahepatic portosystemic shunt for portal hypertension in autosomal recessive polycystic kidney disease. Dig Liver Dis. (2018) 50:707–12. 10.1016/j.dld.2018.03.009 29622386

[8] Carnevale FC Szejnfeld D Moreira AM Gibelli N De Gregorio MA Tannuri U. Long-term follow-up after successful transjugular intrahepatic portosystemic shunt placement in a pediatric patient with Budd-Chiari syndrome. Cardiovasc Intervent Radiol. (2008) 31:1244–8. 10.1007/s00270-008-9400-y 18756372

[9] Ghannam JS Cline MR Hage AN Chick JFB Srinivasa RN Dasika NL. Technical success and outcomes in pediatric patients undergoing transjugular intrahepatic portosystemic shunt placement: a 20-year experience. Pediatr Radiol. (2018). 10.1007/s00247-018-4267-9 30291382

[10] Lorenz JM. Placement of transjugular intrahepatic portosystemic shunts in children. Tech Vasc Interv Radiol/ (2008) 11:235–40. 10.1053/j.tvir.2009.04.007 19527851

[11] Bertino F Hawkins CM Shivaram G Gill AE Lungren MP Reposar A. Technical feasibility and clinical effectiveness of transjugular intrahepatic portosystemic shunt creation in pediatric and adolescent patients. J Vasc Interv Radiol. (2019) 30:178–86.e175. 10.1016/j.jvir.2018.10.003 30717948

[12] Di Giorgio A Agazzi R Alberti D Colledan M D'Antiga L. Feasibility and efficacy of transjugular intrahepatic portosystemic shunt (TIPS) in children. J Pediatr Gastroenterol Nutr. (2012) 54:594–600. 10.1097/MPG.0b013e3182490c05 22228077

[13] Slowik V Monroe EJ Friedman SD Hsu EK Horslen S. Pressure gradients, laboratory changes, and outcomes with transjugular intrahepatic portosystemic shunts in pediatric portal hypertension. Pediatr Transplant. (2019) 23:e13387. 10.1111/petr.13387 30932316

[14] Ghannam JS Cline MR Hage AN Chick JFB Srinivasa RN Dasika NL. Technical success and outcomes in pediatric patients undergoing transjugular intrahepatic portosystemic shunt placement: a 20-year experience. Pediatr Radiol. (2019) 49:128–35. 10.1007/s00247-018-4267-9 30291382

